# Impact of diabetes mellitus and glycemic control on postoperative recurrence of perianal abscess: a retrospective, single-center study

**DOI:** 10.3389/fmed.2026.1758123

**Published:** 2026-01-29

**Authors:** Tingting Li, Jianan Li, Hanwen Yang, Qiang Yu, Yue Wang, Xuecheng Zhang, Xiaoyu Chen

**Affiliations:** Department of Proctology, China-Japan Friendship Hospital, Beijing, China

**Keywords:** diabetes mellitus, glycaemic control, glycated hemoglobin, HbA1c, nomogram, perianal abscess, prediction model, recurrence

## Abstract

**Objective:**

To evaluate the independent effects of diabetes mellitus and the level of glycaemic control on postoperative recurrence of perianal abscess, and to develop and internally validate a clinically applicable risk prediction model.

**Methods:**

We conducted a single-center retrospective cohort study of 232 consecutive adults who underwent primary incision and drainage for perianal abscess between June 2023 and March 2025. Detailed demographic, clinical and operative data were extracted from electronic records, including diabetes status and preoperative glycated hemoglobin (HbA1c). Candidate predictors (age, sex, BMI, lifestyle factors, abscess location, diabetes, HbA1c, inflammatory markers and intraoperative pus residue) were first entered into a LASSO regression for variable selection, followed by multivariable logistic regression. Model performance was assessed in terms of discrimination (area under the receiver operating characteristic curve, AUC), calibration (calibration plots and Hosmer–Lemeshow test) and clinical utility (decision curve analysis), with internal validation by bootstrap resampling (1,000 replicates).

**Results:**

Of the 232 patients (median age 47 years; 53.0% male), 68 (29.3%) experienced recurrence within 6 months. Compared with the non-recurrence group, patients with recurrence had a higher prevalence of diabetes (45.6% vs. 15.2%, *p* < 0.001) and higher median HbA1c levels (9.49% vs. 8.23%, *p* < 0.001), as well as higher age, BMI, a greater proportion of high intersphincteric abscesses and more frequent intraoperative pus residue. Multivariable analysis identified five independent predictors of recurrence: diabetes mellitus (OR 3.99, 95% CI 1.61–9.88, *p* = 0.003), HbA1c level (OR 1.50 per 1% increase, 95% CI 1.18–1.92, *p* = 0.001), BMI (OR 1.72 per kg/m^2^, 95% CI 1.43–2.06), age (OR 1.11 per year, 95% CI 1.06–1.16, *p* < 0.001), and low versus high intersphincteric abscess location (OR 0.36, 95% CI 0.14–0.97, *p* = 0.044). The resulting prediction model achieved an AUC of 0.897 (95% CI 0.814–0.957); after bootstrap internal validation, the optimism-corrected Harrell’s C-index was 0.892. Calibration plots showed good agreement between predicted and observed recurrence probabilities, and decision curve analysis indicated a consistent net clinical benefit across a wide range of threshold probabilities.

**Conclusion:**

Diabetes mellitus and inadequate glycaemic control are important, independent risk factors for postoperative recurrence of perianal abscess, with a clear dose–response relationship between HbA1c and recurrence risk. The internally validated prediction model, which combines HbA1c with other readily available clinical variables, shows promise as a tool for early identification of high-risk patients and may support more personalized perioperative optimization and follow-up. External validation and impact studies are required before routine implementation in diverse clinical settings.

## 1 Introduction

Perianal abscess is a common acute suppurative infection arising from the anal glands and crypts and represents one of the most frequent emergencies in colorectal surgical practice (1, 2). Although prompt incision and drainage (I&D) usually relieve the acute episode, reported recurrence rates range from approximately 10% to over 40%, and a considerable proportion of patients ultimately develop complex anal fistula requiring repeated operations and prolonged follow-up (3). Thus, resolution of the initial abscess does not necessarily equate to cure but rather marks the beginning of a chronic disease trajectory for a substantial subset of patients. This high burden of recurrence and progression underscores an important unmet need for early risk stratification and targeted preventive strategies.

The pathogenesis of recurrent perianal abscess is multifactorial, involving local anatomical, microbiological and systemic host factors. Inadequate drainage of the primary cavity, missed fistulous tracts and persistent infected anal glands are recognized surgical contributors (4). The polymicrobial flora, typically comprising gut-derived aerobes and anaerobes, further complicates eradication of infection (5). However, the host’s systemic capacity for immune defense and wound healing may be the most crucial determinant of postoperative outcomes. Among systemic comorbidities, diabetes mellitus (DM) is particularly relevant because of its well-described effects on innate immunity, microvascular perfusion and tissue repair. Epidemiological studies have demonstrated a strong association between DM and perianal sepsis: individuals with diabetes have a higher risk of developing perianal abscess, and perianal sepsis may occasionally be the sentinel event that leads to the diagnosis of previously unrecognized diabetes (6, 7). Mechanistically, chronic hyperglycemia impairs neutrophil function, reduces phagocytic capacity and promotes microangiopathy, resulting in tissue hypoxia and delayed wound healing (8–10). Diabetic neuropathy may also blunt pain perception and delay presentation in the event of recurrence (11). These pathophysiological changes create a permissive environment for persistent infection and repeated abscess formation.

Despite the acknowledged link between DM and perianal abscess, important gaps remain in our understanding of how disturbances in glucose metabolism influence the risk of postoperative recurrence. Most previous studies have dichotomized DM as present or absent, which fails to capture the continuous spectrum of dysglycemia and the potential dose–response relationship between the degree of glycemic control and recurrence risk. Glycated hemoglobin (HbA1c) provides an objective measure of chronic glycemic control and is a modifiable factor that may refine prognostication beyond a simple diabetes diagnosis. Although poor glycemic control has been associated with adverse surgical outcomes across various specialties (12, 13), its specific quantitative relationship with perianal abscess recurrence has not been clearly defined. Furthermore, existing studies have rarely integrated HbA1c with other clinical and operative variables into a comprehensive prediction model, and to our knowledge no validated risk score specifically targeting recurrence after perianal abscess drainage is currently available. Consequently, clinicians lack an evidence-based tool to identify high-risk patients at the time of initial treatment.

Health inequalities related to perianal sepsis have been only partially explored. Limited evidence suggests that age, sex and socioeconomic status may influence disease incidence and access to timely surgical care, but robust data on their impact on recurrence and fistula formation are scarce. In addition, potential differences across sociodemographic groups in the prevalence and control of DM could further modify recurrence risk. Clarifying the role of these sociodemographic factors, alongside metabolic and surgical variables, is important to ensure that any prediction model is applicable across diverse patient populations and does not inadvertently exacerbate existing disparities in outcomes.

In this context, we conducted a retrospective cohort study of adult patients undergoing surgical I&D for primary perianal abscess at a tertiary care center. Our first objective was to evaluate the independent associations of diabetic status and preoperative HbA1c level with postoperative recurrence of perianal abscess, while adjusting for a broad range of demographic, sociodemographic, clinical and operative covariates. Our second objective was to develop and internally validate a clinically applicable prediction model, presented as a nomogram, that combines glycemic parameters with other key predictors to estimate the individual risk of recurrence. The model is intended for use by colorectal surgeons and emergency physicians during the preoperative assessment and early postoperative period to guide patient counseling, optimization of glycemic control and tailoring of follow-up strategies.

## 2 Methods

### 2.1 Study design and ethical considerations

This investigation was conceived as a single-center, retrospective observational cohort study, conducted within the Department of Proctology at the China-Japan Friendship Hospital, a tertiary referral center. The study protocol received full approval from the Hospital’s Institutional Review Board prior to commencement (Approval No. 2023-KY-363-1). Given the retrospective nature of the research, which involved the analysis of pre-existing, anonymized clinical data, the requirement for obtaining individual informed consent was formally waived by the ethics committee. The study was conducted in strict adherence to the ethical principles outlined in the Declaration of Helsinki. A sample size calculation was performed prior to data collection using G*Power software (version 3.1.9.7, Universität Düsseldorf, Germany). Based on an estimated recurrence rate of 25%, a two-tailed alpha of 0.05, and power of 80%, the minimum required sample size to detect a medium effect size (odds ratio ~1.8) in logistic regression with up to 5 predictors was 191 patients. Therefore, the final sample of 232 patients was considered adequate to support valid statistical inferences.

### 2.2 Study population and eligibility criteria

The study cohort was derived from a comprehensive screening of all consecutive adult patients (age ≥ 18 years) who underwent surgical intervention for a primary perianal abscess at our institution between June 2023 and March 2025.

Patient eligibility was rigorously assessed against predefined criteria.

Inclusion criteria were as follows:

- First-time diagnosis of perianal abscess, confirmed intraoperatively by the definitive finding of purulent discharge.
- Treated with initial incision and drainage procedure.
- Availability of a documented preoperative glycated hemoglobin (HbA1c) level measured within 30 days prior to surgery.

Exclusion criteria included:

- Perianal abscess secondary to specific underlying diseases (e.g., Crohn’s disease, ulcerative colitis, tuberculosis, or malignancy).
- History of previous anorectal surgery for abscess or fistula.
- Presentation with a recurrent abscess at the index operation.
- Pregnancy or lactation.
- Incomplete medical records.
- Loss to follow-up before the 6-month outcome assessment.

### 2.3 Data collection and variable definitions

All variables were checked for completeness and plausibility before analysis. Cases with missing data on key predictors or outcomes were excluded from the analysis (no imputation was performed). Data cleaning and preprocessing procedures were applied uniformly across all sociodemographic subgroups. Most exclusions due to incomplete records were related to missing preoperative HbA1c measurements or absence of sufficiently detailed follow-up documentation to ascertain recurrence status.

A meticulous process of data extraction was undertaken by two trained clinical researchers who were blinded to the study’s primary hypothesis to minimize ascertainment bias. Data were sourced from electronic medical records, anesthesia charts, operative notes, and laboratory systems using a standardized, pre-piloted data collection form. Any discrepancies in data extraction were resolved through consensus or by adjudication from a senior colorectal surgeon. During data abstraction, the two clinical researchers were not informed about the recurrence status of individual patients, so the recording of baseline predictors was effectively blinded to the outcome.

The primary outcome of this study was postoperative recurrence of perianal abscess, explicitly defined as the clinical or radiological diagnosis of a new abscess occurring in the same perianal region, which subsequently required a repeat surgical drainage procedure within the 6-month period following the initial operation. This definition aligns with established clinical endpoints in the literature (12). Both abscess location and intraoperative assessment of pus residue were determined and documented intraoperatively by board-certified colorectal surgeons or senior residents under specialist supervision, representing the usual specialist workforce of our tertiary proctology center.

The primary exposures of interest were the presence of DM and the degree of Glycemic Control.

A patient was classified as having DM if there was a documented physician diagnosis of type 1 or type 2 DM in their medical history or if they were actively prescribed anti-diabetic medication (oral agents or insulin), irrespective of the preoperative HbA1c level. Glycemic control was quantitatively represented by the continuous variable of the preoperative HbA1c level (%).

A comprehensive set of covariates was collected to account for potential confounding. These included baseline demographic characteristics (age, sex, Body Mass Index (BMI)), lifestyle factors (smoking status categorized as “current” or “non-smoker”; alcohol consumption categorized as “regular” or “non-drinker” based on standardized units per week), and abscess-specific characteristics. The anatomical location of the abscess was classified based on a synthesis of preoperative imaging (MRI when available) and detailed intraoperative findings according to the standardized Parks’ classification system, later simplified to a binary variable (“High Intersphincteric” or “Low Intersphincteric”) for analytical purposes. A critical intraoperative variable, the presence of Pus Residue, was recorded as a binary (Yes/No) outcome based on the operating surgeon’s explicit documentation regarding the completeness of drainage, specifically noting any inadequately drained loculations or residual purulent material. Laboratory parameters, namely the preoperative White Blood Cell (WBC) count and Neutrophil Percentage (NEUT%), were retrieved from the complete blood count test performed closest to the surgery date.

Surgical technique and postoperative antibiotic use were considered part of standard care and were not included as predictors in the model, whereas the intraoperative assessment of pus residue was captured as a binary predictor.

A 6-month time horizon was chosen because previous studies and our institutional experience indicate that the vast majority of clinically relevant recurrences occur within this period. The same definition of recurrence and follow-up schedule was applied uniformly to all patients irrespective of age, sex, or other sociodemographic characteristics.

To promote fairness, identical inclusion criteria, data collection procedures, and modeling steps were applied to all patients irrespective of age, sex, or other sociodemographic characteristics. Age and sex were included among the candidate predictors to account for potential baseline differences between sociodemographic groups; no additional algorithmic fairness constraints were imposed given the modest sample size and single-center design.

### 2.4 Surgical management and postoperative follow-up protocol

All surgical procedures were performed by attending colorectal surgeons or senior residents under their direct supervision. The standard of care involved an incision and drainage procedure conducted under spinal or general anesthesia. A radial incision was made over the point of maximal fluctuation, followed by thorough evacuation of pus, deliberate breaking down of all loculations, and ensuring adequate drainage of the abscess cavity. The decision to perform concomitant fistula exploration or to place a seton was made intraoperatively at the surgeon’s discretion, based on the identification of a definite internal opening or the concern for a complex fistula.

Postoperative management adhered to a standardized institutional protocol, which included daily wound care with packing, regular sitz baths, and appropriate analgesia. The administration of postoperative antibiotics was not uniform but was instituted in cases with signs of systemic infection or extensive surrounding cellulitis. A structured follow-up protocol was mandated for all patients, with scheduled outpatient clinic visits at 1 week, 1 month, 3 months, and 6 months post-surgery. During these visits, a physical examination of the wound was performed to assess healing and identify any signs of recurrence. For patients who failed to attend their scheduled appointments, a structured telephone interview was conducted by a research nurse to systematically ascertain their clinical status and specifically inquire about symptoms suggestive of recurrence. Patients were asked about key clinical indicators including persistent or worsening pain, swelling, purulent discharge, fever, and any urgent care visits or repeat surgical interventions since their index operation. Only telephone assessments that yielded clear and consistent information regarding absence or presence of recurrence, based on predefined criteria, were accepted; cases with ambiguous or insufficient information were classified as not meeting follow-up standards and were excluded from outcome analysis. Outcome assessments were therefore performed by board-certified colorectal surgeons or senior surgical residents under specialist supervision during clinic visits, and by a trained research nurse using a standardized questionnaire during telephone follow-up; all assessors were staff members of the same tertiary proctology center.

Because this was a retrospective cohort and the outcome occurred after the baseline assessment, no additional blinding procedures were implemented for outcome assessment beyond routine clinical practice. At the time of data abstraction, the recurrence status of individual patients was obtained from the medical records after all baseline predictor data had been collected, and was not influenced by knowledge of the planned prediction model.

### 2.5 Statistical analysis

The study sample size was determined pragmatically by the number of consecutive eligible patients treated at our institution during the predefined study period; no formal *a priori* sample size calculation was performed. For prediction modeling, we considered events-per-variable recommendations. With 68 recurrence events available and a final model including five predictors, the effective number of events per predictor was approximately 13, which is generally regarded as adequate for logistic regression–based prediction models. Although the proportion of recurrence events (68/232, 29.3%) indicated some degree of class imbalance between outcome categories, this imbalance was not extreme. We therefore did not apply additional resampling or class-weighting techniques. The risk of overfitting and potential impact of outcome prevalence were instead mitigated through the use of penalized regression (LASSO) and bootstrap-based internal validation.

All data for model development and internal validation were derived from this single retrospective cohort of consecutive patients, which is considered representative of adults treated for primary perianal abscess at tertiary proctology centers. The cohort was not randomly partitioned into separate training and testing subsets; instead, the entire dataset was used for model development, and model optimism was quantified using bootstrap resampling for internal validation in accordance with current prediction-modeling guidance for moderately sized datasets. All patients were treated at a single tertiary center and no clustering structure (such as hospitals or geographical regions) was present; therefore, multilevel modeling and between-cluster heterogeneity in predictor effects were not evaluated. No model updating procedures (such as recalibration or revision of predictor effects) were performed after internal validation; the coefficients from the original multivariable logistic regression model were retained for the final nomogram.

All statistical computations were executed using the R software environment (version 4.2.0; R Foundation for Statistical Computing, Vienna, Austria). A two-tailed *p*-value of less than 0.05 was designated as the threshold for statistical significance. The initial phase of analysis involved comprehensive descriptive statistics. The normality of continuous variables was formally assessed using the Shapiro–Wilk test and visual inspection of Q–Q plots. Accordingly, normally distributed data are summarized as mean ± standard deviation (SD) and inter-group comparisons were made using the independent-samples *t*-test. Non-normally distributed data are presented as median with interquartile range (IQR), and the Mann–Whitney U test was employed for comparisons. Categorical variables are expressed as frequencies and percentages, with inter-group differences assessed using the chi-square test or Fisher’s exact test, as appropriate.

Continuous predictors (age, BMI, HbA1c, preoperative WBC count, and neutrophil percentage) were entered into the model on their original scales without centering or standardization in order to preserve clinical interpretability, and no *a priori* categorization or transformation was applied. Categorical predictors (sex, smoking status, alcohol consumption, abscess location, intraoperative pus residue, and diabetes status) were coded as binary indicator variables. Given the limited number of outcome events, interaction terms between predictors were not included in the primary model. All candidate predictors were specified *a priori* based on existing literature on risk factors for perianal sepsis and on clinical expertise, and no additional data-driven pre-selection was performed before model building.

Because the outcome of interest was binary and we aimed to obtain both risk estimates and clinically interpretable effect sizes, multivariable logistic regression was chosen as the modeling framework. Given the relatively high number of candidate predictor variables relative to the number of outcome events, we utilized the LASSO regression method with 10-fold cross-validation to penalize coefficients and select the most robust predictors for the multivariable model from the initial set of candidates (age, sex, BMI, smoking, alcohol, abscess location, diabetes status, HbA1c, WBC, neutrophil percentage, and pus residue). The optimal tuning parameter (*λ*) was selected based on the value that minimized the binomial deviance (lambda.min). The predictors retained by the LASSO procedure (those with non-zero coefficients) were then entered into a multivariable binary logistic regression model to ascertain their independent association with the risk of recurrence, with results expressed as adjusted odds ratios (ORs) and their corresponding 95% confidence intervals (CIs).

For each patient, an individual predicted probability of postoperative recurrence was obtained by multiplying the estimated regression coefficients by the corresponding predictor values to derive a linear predictor (*η*), which was then transformed using the logistic function \(*p* = 1/(1 + \exp.(−\eta))\). These predicted probabilities were used to generate the receiver operating characteristic (ROC) curve, calibration plot, decision curve, and the graphical nomogram. We did not predefine fixed probability thresholds to classify patients into discrete low- or high-risk categories; instead, clinically relevant thresholds can be chosen by clinicians based on specific decision contexts, guided by the trade-offs illustrated in the decision curve analysis.

In exploratory analyses, we also assessed model performance in key sociodemographic subgroups defined by sex (male vs. female) and age (≤50 vs. >50 years) to explore the stability of discrimination and calibration across these strata. The performance of this prediction model was rigorously evaluated across three key domains: discrimination, calibration, and clinical utility. Discrimination, the model’s ability to differentiate between patients with and without recurrence, was quantified by the area under the ROC curve (AUC). Calibration, which reflects the agreement between predicted probabilities and observed frequencies of recurrence, was assessed visually using a calibration plot and supplemented with the Hosmer–Lemeshow goodness-of-fit test. The clinical net benefit of applying the model across a range of decision thresholds was evaluated using decision curve analysis (DCA). Finally, to account for inherent overoptimism and internally validate the model’s performance, bootstrap resampling with 1,000 replicates was performed, and a bias-corrected Harrell’s C-index was calculated and reported.

In the final multivariable logistic regression model, the log-odds of postoperative recurrence for an individual patient are given by:


$$\text{logit}(p)=\beta 0+0.104\times \mathrm{Age}\;(\text{years})+0.542\times \mathrm{BMI}\;(\mathrm{kg}/m^{2})$$

−1.022×I_low_intersphincteric

+0.928×I_pus_residue

+1.384×I_diabetes

+0.405×HbA1c(%)

where I_low_intersphincteric, I_pus_residue, and I_diabetes are indicator variables taking the value 1 if the patient has a low intersphincteric abscess, intraoperative pus residue present, or diabetes mellitus, respectively, and 0 otherwise.

## 3 Results

### 3.1 Baseline characteristics

The study cohort of 232 patients was stratified into 164 non-recurrence and 68 recurrence cases. Comparative analysis demonstrated significant disparities in key clinical features, with the recurrence cohort presenting at an advanced age, with elevated BMI, a higher frequency of high intersphincteric abscesses, and increased incidence of intraoperative pus residue (all *p* < 0.01). Furthermore, both the prevalence of diabetes and the median HbA1c level were significantly different (both *p* < 0.001), while no differences were observed between the two groups regarding sex distribution, smoking or alcohol consumption status, or preoperative inflammatory markers (Table 1).

**Table 1:** Baseline characteristics.

Character	Non-recurrence *N* = 164	Recurrence *N* = 68	*p* value
Age (Years old)	45 (38, 52)	51 (45, 59)	<0.001^a^
Gender, *n* (%)			0.784^b^
Female	78 (47.6%)	31 (45.6%)	
Male	86 (52.4%)	37 (54.4%)	
BMI (kg/m^2^)	25.5 (23.3, 27.4)	28.9 (26.7, 31.1)	<0.001^a^
Smoking, *n* (%)			0.438^b^
No	125 (76.2%)	55 (80.9%)	
Yes	39 (23.8%)	13 (19.1%)	
Drinking, *n* (%)			0.397^b^
No	129 (78.7%)	50 (73.5%)	
Yes	35 (21.3%)	18 (26.5%)	
Abscess space, *n* (%)			0.002^b^
High bit	24 (14.6%)	22 (32.4%)	
Low bit	140 (85.4%)	46 (67.6%)	
Intraoperative pus residue, *n* (%)			<0.001^b^
No	143 (87.2%)	45 (66.2%)	
Yes	21 (12.8%)	23 (33.8%)	
DM, *n* (%)			<0.001^b^
No	139 (84.8%)	37 (54.4%)	
Yes	25 (15.2%)	31 (45.6%)	
Hba1c (%)	8.23 (7.46, 8.99)	9.49 (7.66, 11.23)	<0.001^a^
ba1c_category, *n* (%)			0.006^b^
<7.5%	44 (26.8%)	7 (10.3%)	
>7.5%	120 (73.2%)	61 (89.7%)	
Preoperative WBC	9.7 (7.0, 11.6)	9.6 (6.8, 11.9)	0.722^a^
Preoperative NEUT	69 (63, 77)	67 (61, 74)	0.295^a^

a Wilcoxon rank sum test.

b Pearson’s Chi-squared test.

All 232 eligible patients with complete baseline and 6-month outcome information were included in the analysis; no additional exclusions occurred during follow-up.

### 3.2 Prediction model

**Figure 1:**
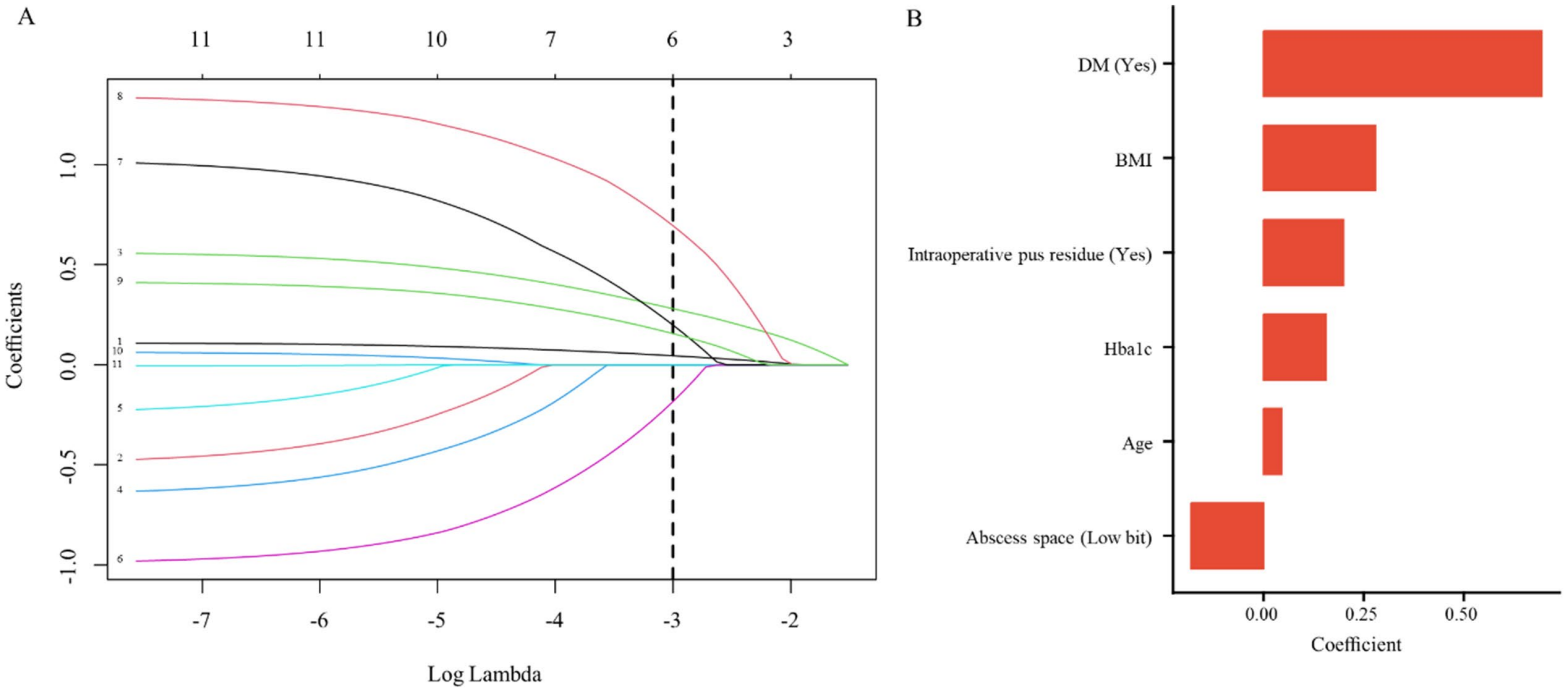
Variable selection path **(A)** and coefficients **(B)** from LASSO regression.

All 232 patients (including 68 recurrence events) were entered into the LASSO procedure and subsequent multivariable logistic regression model; no additional exclusions were made for the modeling analyses. Candidate predictor variables included age, sex, BMI, smoking status, alcohol consumption status, abscess location, diabetes status, glycated HbA1c, preoperative white blood cell count, preoperative neutrophil percentage, and intraoperative pus residue. These variables were incorporated into the initial model, following which LASSO regression analysis was applied. This process ultimately identified six potential predictors: DM, BMI, intraoperative pus residue, HbA1c, Age, and abscess location. The variable selection path plot and the regression coefficients derived from the LASSO analysis are illustrated in Figures 1A,B, respectively.

**Figure 2:**
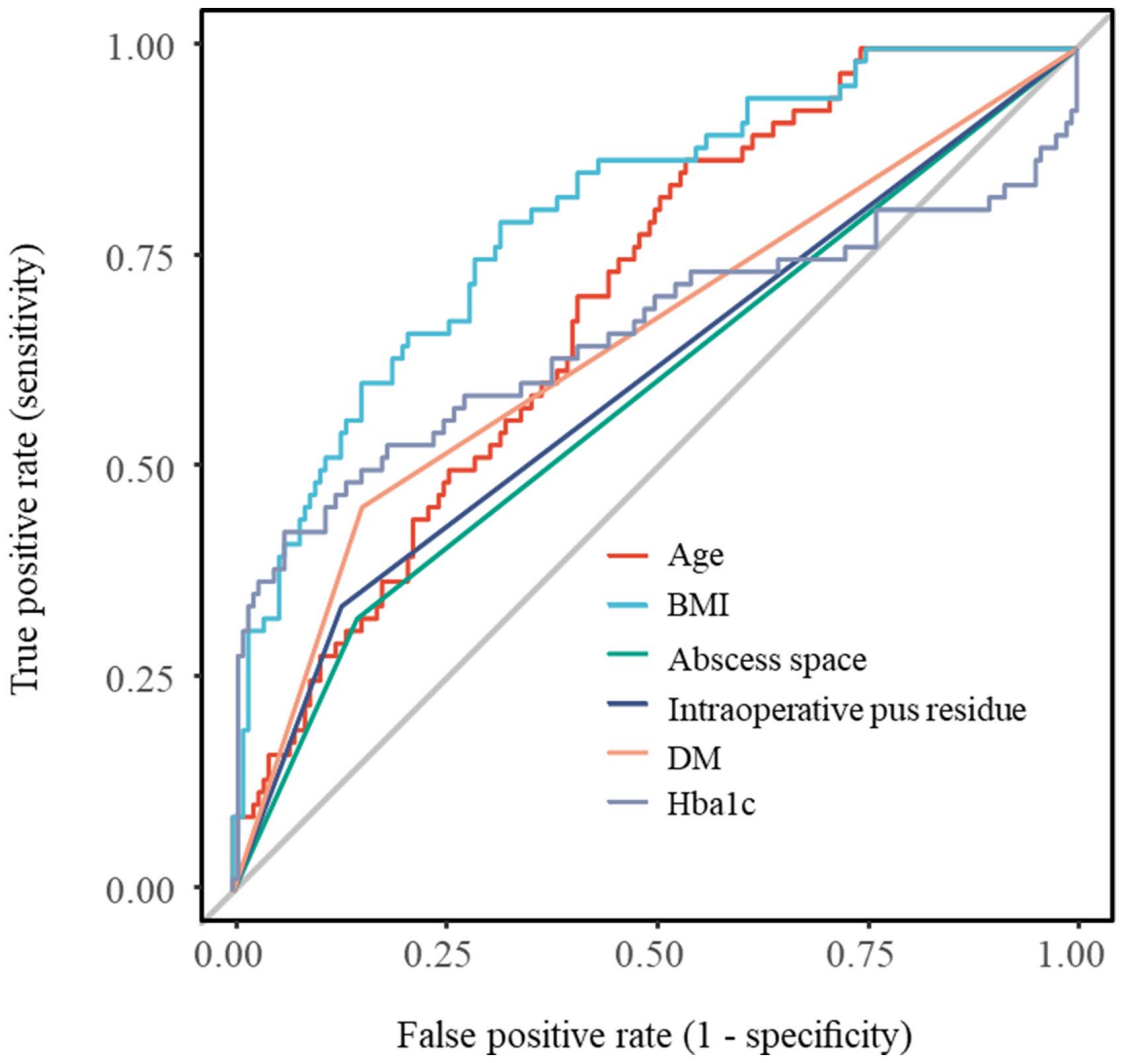
Comparison of ROC curves for individual predictor variables.

The predictive capacity of each individual factor was evaluated using univariate logistic regression, with the resulting ROC curves presented in Figure 2. ROC curve analysis demonstrated that age (AUC = 0.699, 95% CI: 0.630–0.768), BMI (AUC = 0.803, 95% CI: 0.742–0.865), abscess location (AUC = 0.589, 95% CI: 0.526–0.651), intraoperative pus residue (AUC = 0.605, 95% CI: 0.543–0.667), diabetes (AUC = 0.652, 95% CI: 0.586–0.717), and HbA1c (AUC = 0.658, 95% CI: 0.566–0.751) all possessed a certain degree of predictive ability for the outcome event. Among these, BMI exhibited the highest discriminative performance.

**Figure 3:**
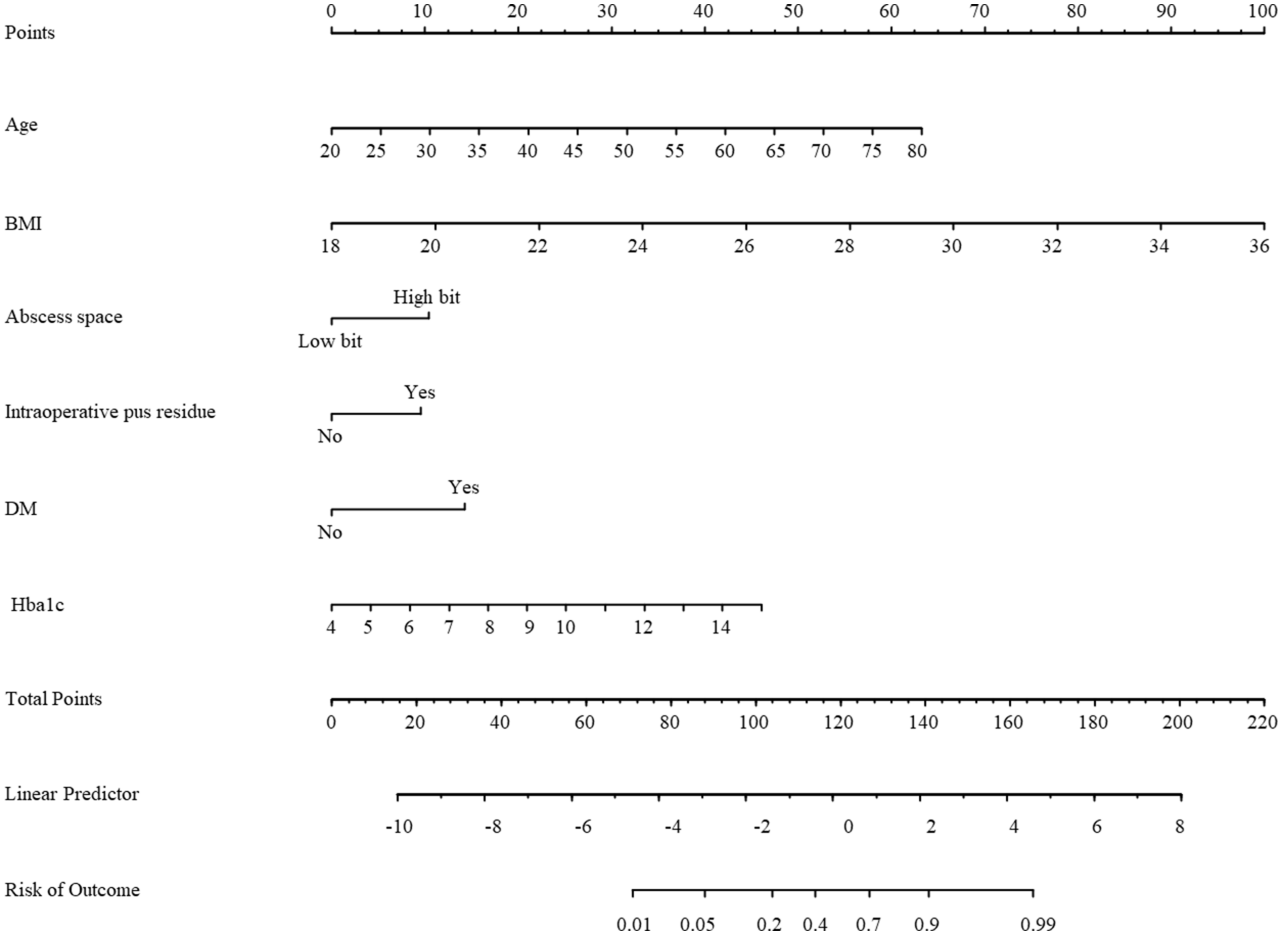
Nomogram of the prediction model.

**Figure 4:**
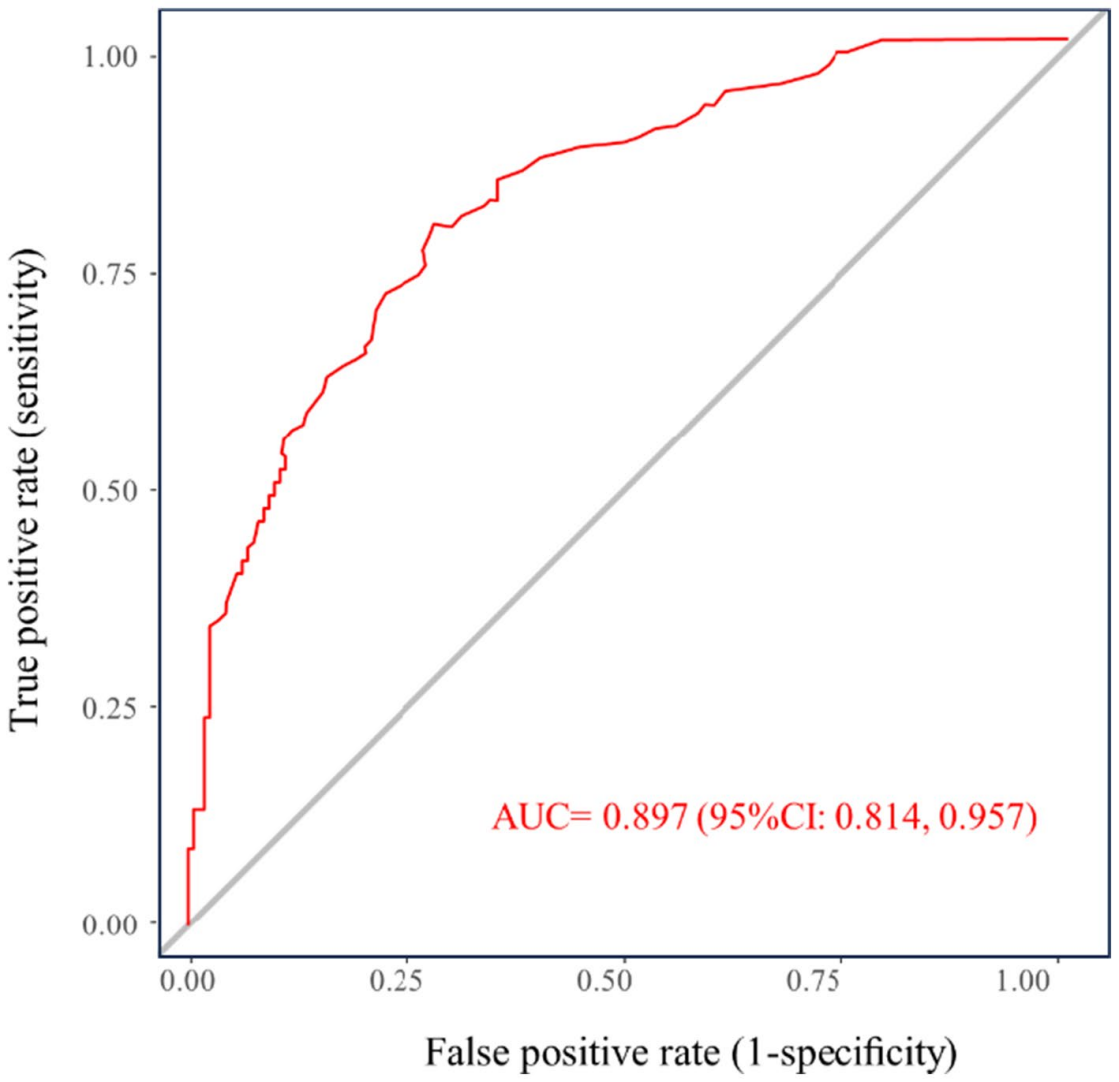
ROC curve of the prediction model.

To ascertain independent determinants of recurrence, a multivariable logistic regression model was subsequently developed incorporating the predictors identified through LASSO regression (Table 2). The final model confirmed several significant risk factors: advanced age (*p* < 0.001), elevated BMI (*p* < 0.001), and higher HbA1c levels (*p* = 0.001) demonstrated statistically significant associations with increased recurrence risk. Additionally, the presence of diabetes mellitus was identified as a strong independent predictor (*p* = 0.003). In terms of anatomical characteristics, abscesses located in the low intersphincteric space were associated with a substantially reduced risk compared to those in the high location (*p* = 0.044). While intraoperative pus residue showed a clinically relevant trend toward increased recurrence risk, this association did not reach statistical significance in the adjusted model (*p* = 0.078). Based on the variables identified in the multivariable logistic regression analysis, a simplified nomogram was constructed, as illustrated in Figure 3. The predictive performance of the model was evaluated using the ROC curve, yielding an AUC of 0.897 (95% CI: 0.814, 0.957). The ROC curve of the prediction model is presented in Figure 4.

**Table 2:** Multivariable logistic regression model for postoperative recurrence.

Characteristic	Beta_coefficient	Standard_error	OR	CI_lower	CI_upper	p_value
Intercept	β0 (from R output)	SE0 (from R output)				
Age (years)	0.104	0.023	1.11	1.06	1.16	<0.001
BMI (kg/m^2^)	0.542	0.093	1.72	1.43	2.06	<0.001
Abscess space – High intersphincteric			1			
Abscess space – Low intersphincteric	−1.022	0.494	0.36	0.14	0.97	0.044
Intraoperative pus residue – No			1			
Intraoperative pus residue – Yes	0.928	0.527	2.53	0.9	7.09	0.078
Diabetes mellitus – No			1			
Diabetes mellitus – Yes	1.384	0.463	3.99	1.61	9.88	0.003
HbA1c (%)	0.405	0.124	1.5	1.18	1.92	0.001

### 3.3 Validation and calibration

**Figure 5:**
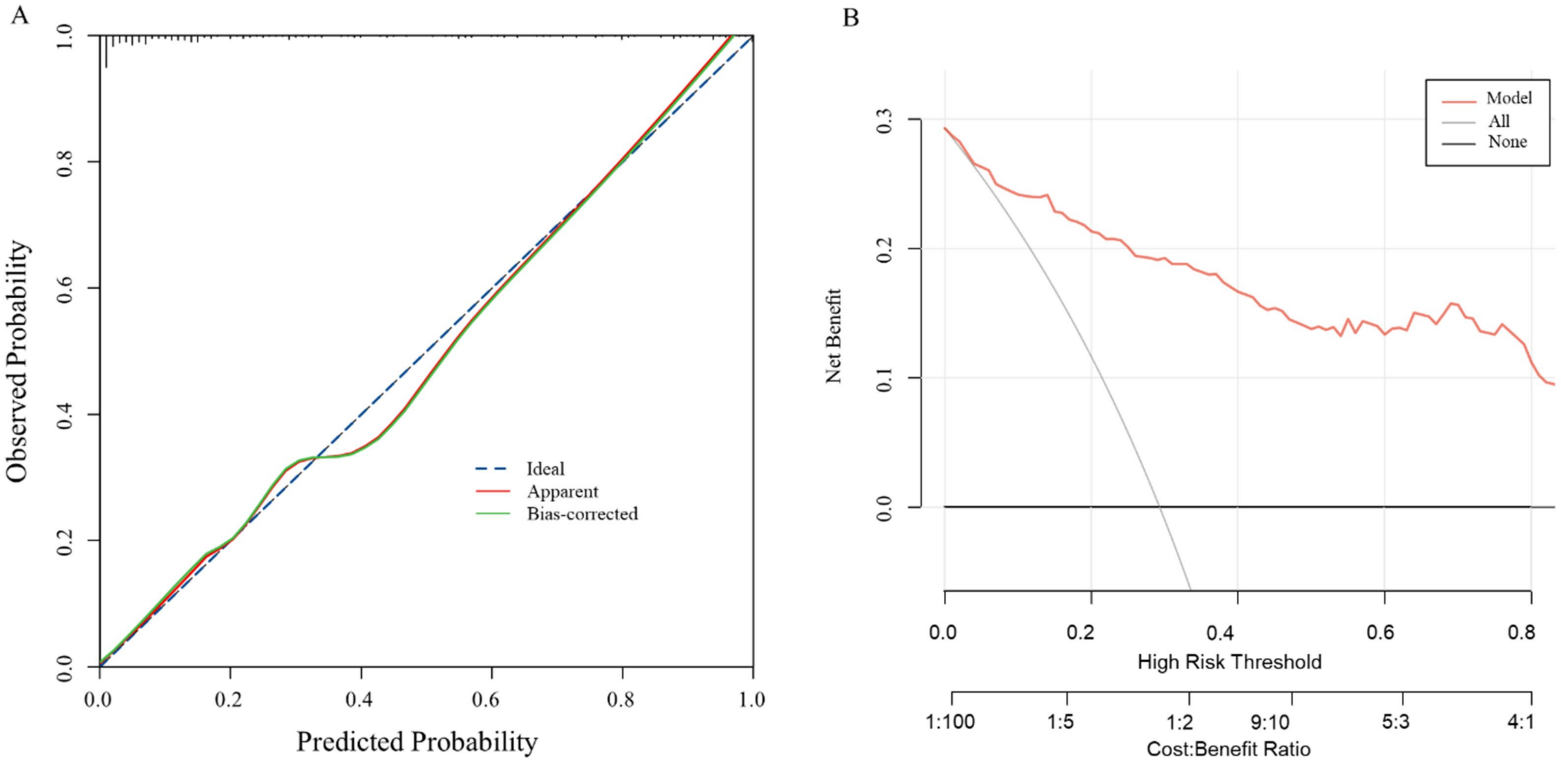
Calibration curve **(A)** and decision curve analysis **(B)** of the model.

Internal validation, performed via 1,000 bootstrap replicates, confirmed the model’s robustness and discriminative capacity, yielding a bias-corrected Harrell’s C-index of 0.892. The calibration curve for the nomogram (Figure 5A) demonstrated high consistency between the observed outcomes and the predicted probabilities of perianal abscess recurrence. The calibration plot closely adhered to the ideal 45-degree diagonal line, indicating excellent agreement between predictions and observations. The clinical practicality of the nomogram was affirmed by its robust net benefit across plausible probability thresholds, as evidenced by the DCA in Figure 5B, supporting its integration into clinical practice.

Exploratory subgroup analyses suggested that the model retained good discrimination and acceptable calibration across key sociodemographic strata. Among male patients, the AUC for predicting recurrence was 0.90 (95% CI 0.82–0.97), with a calibration curve closely following the 45-degree line. In female patients, the AUC was 0.88 (95% CI 0.75–0.96), and visual inspection likewise showed good agreement between predicted and observed risks.

**Figure 6:**
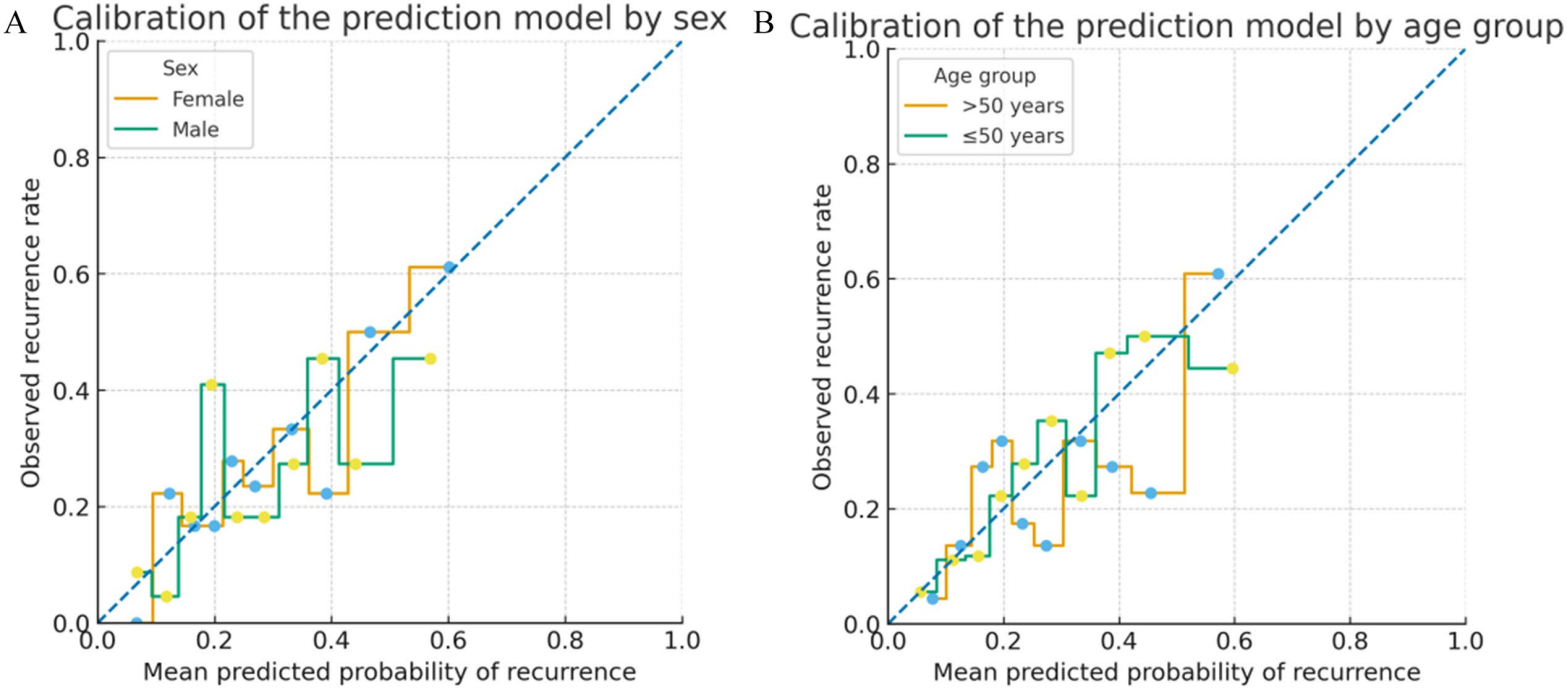
Subgroup calibration plots of the prediction model. **(A)** Calibration of the prediction model in male and female patients. Predicted probabilities of recurrence were grouped into deciles within each subgroup, and the observed event rates in each decile are shown as a stepwise function against the mean predicted risk. The dashed diagonal line represents perfect calibration. **(B)** Calibration of the prediction model in patients aged ≤50 years and >50 years, using the same decile-based stepwise approach. In all subgroups, the observed risks closely followed the 45-degree line, indicating acceptable calibration across key sociodemographic strata.

When stratified by age, the AUC was 0.89 (95% CI 0.80–0.96) in patients aged ≤50 years and 0.89 (95% CI 0.79–0.96) in those older than 50 years, again with no major deviations from perfect calibration in either subgroup. Decision curve analyses by sex and age subgroups (data not shown) were qualitatively similar to those for the overall cohort, indicating consistent net clinical benefit across these sociodemographic groups (Figure 6).

## 4 Discussion

This study provides strong evidence that diabetes status and, more critically, the degree of glycaemic control are independent determinants of postoperative recurrence following incision and drainage for perianal abscess. Our findings reveal a dose–response relationship between preoperative HbA1c levels and recurrence risk, demonstrating that each 1% increase in HbA1c is associated with a 50% increase in the odds of recurrence. This insight moves beyond a binary classification of diabetes and establishes glycaemic control as a continuous risk spectrum, underscoring that the quality of metabolic management is as critical as the diagnosis itself. The prediction model we developed, which integrates these glycaemic parameters with other clinical variables, showed excellent apparent and internally validated performance (AUC 0.897), offering a potentially useful tool for individualized risk assessment.

The strong associations observed are biologically plausible and consistent with the known pathophysiological consequences of chronic hyperglycaemia. The significantly higher median HbA1c in the recurrence group (9.49% vs. 8.23%) signifies a state of persistent immune dysfunction that operates through several interconnected pathways. Hyperglycaemia induces a functional immunodeficiency by impairing key neutrophil functions, including chemotaxis, phagocytosis, and intracellular killing, thereby weakening the primary cellular defense against residual pathogens (8, 9). This is compounded by defects in humoral immunity and complement system activation. In parallel, diabetes-induced microangiopathy and endothelial dysfunction compromise local tissue perfusion and oxygen delivery, creating a hypoxic and nutrient-deprived wound environment that hinders fibroblast proliferation, collagen synthesis, and epithelialisation, thereby delaying tissue repair (10, 11). The identification of diabetes as an independent predictor (OR = 3.99) even after adjustment strongly suggests that these systemic impairments create a permissive milieu for subclinical infection to persist and eventually manifest as clinical recurrence. This triad—immune dysfunction, microvascular insufficiency, and impaired healing—provides a coherent mechanistic framework that is captured by our statistical model.

Our results corroborate and extend previous work. The pivotal role of diabetes aligns with findings by Sarofim et al. (12) and Qiao et al. (13), who identified it as a key predictor for poor outcomes. Adamo et al. (14) demonstrated in a population-based study that poor glycaemic control amplifies the risk of developing perianal abscess in diabetic patients. Our study builds upon this foundation by demonstrating that the same principle of glycaemic control critically influences the postoperative course, specifically recurrence. Importantly, we quantified the risk associated with the continuum of glycaemic control, a dimension often overlooked, thereby emphasizing that risk stratification should not stop at the diagnosis of diabetes but must incorporate HbA1c levels. This observation is consistent with surgical literature from other disciplines, where elevated preoperative HbA1c is linked to increased complications in cardiac, orthopedic, and general surgery (15–17). The central role of glycaemic control is further underscored by Dong et al. (18), who also identified elevated HbA1c as a key factor influencing prognosis after perianal abscess surgery, a conclusion that our model supports. The confirmation of elevated BMI as one of the strongest predictors (OR = 1.72) reinforces the well-established link between obesity and impaired wound healing, likely mediated by adipose tissue–derived chronic inflammation, relative tissue hypoxia, and technical challenges in the deep pelvic region (19, 20). The protective effect associated with a low intersphincteric abscess location (OR = 0.36) highlights the importance of anatomical complexity, with high abscesses more likely to be associated with deeper, inadequately drained infectious sources and a higher probability of an occult trans-sphincteric fistula, which itself is a risk factor for recurrence. The non-significant trend for intraoperative pus residue (OR = 2.53, *p* = 0.078) nonetheless points toward the critical importance of surgical technique and complete source control. This technical challenge is particularly relevant in complex or recurrent cases, as illustrated by Zhang et al. (21) in their case report, which emphasizes that meticulous management of the fistula tract is essential to prevent recurrence. Our findings translate this case-level insight into quantitative, population-level evidence.

The developed nomogram, with its high discriminative ability and good calibration, offers a practical way to move from reactive to proactive patient management. Its clinical utility, as demonstrated by decision curve analysis, lies in enabling early identification of high-risk individuals at the point of care. From a usability perspective, the nomogram can be implemented as a simple paper tool or electronic calculator and is intended for use by colorectal surgeons, proctologists, and other clinicians familiar with perianal sepsis; no advanced statistical expertise is required for routine application. Because the model requires all five predictors, risk calculation is not recommended when key inputs such as preoperative HbA1c are unavailable or of doubtful quality; in such situations, clinicians should rely on conventional clinical judgement rather than extrapolating model-based estimates. We envisage that, in settings where routine HbA1c testing is feasible, the nomogram could support a risk-stratified management strategy. For patients identified as high risk, a bundle of intensified interventions may be appropriate: (1) preoperative optimization of metabolic status where time allows, ideally with endocrine consultation and efforts to improve glycaemic control; (2) enhanced intraoperative vigilance by experienced surgeons, including meticulous exploration for fistulous tracts, thorough curettage of the abscess cavity, and consideration of fistulotomy or seton placement when an internal opening is suspected; and (3) more intensive postoperative surveillance, with closer follow-up, a lower threshold for postoperative imaging, and reinforced patient education on wound care and glycaemic monitoring. These proposals are hypothesis-generating and should be formally evaluated in prospective studies before being adopted as standard care.

This study also has limitations that must be considered when interpreting the findings. First, the retrospective, single-center design carries risks of selection bias and unmeasured confounding (e.g., socioeconomic status, detailed medication adherence) and may limit generalisability beyond similar tertiary proctology centers. Second, although the effective number of events per predictor was acceptable and penalized regression with bootstrap internal validation was used, overfitting cannot be completely excluded; the high C-index should therefore be interpreted cautiously until external validation in independent cohorts is available. Third, a complete-case analysis was performed, with patients lacking key predictors or outcome data excluded; while this approach enhanced internal consistency, it may have introduced some degree of selection bias. Fourth, data on specific microbiological profiles and detailed antibiotic regimens were unavailable, which could provide further insights into the mechanisms linking hyperglycaemia to recurrence—for example, whether diabetic patients harbor more virulent or resistant microbial flora (22). Fifth, the definition of “intraoperative pus residue,” although clinically intuitive, contains a subjective component and is susceptible to inter-observer variation. Finally, follow-up was limited to 6 months; although most recurrences are likely to occur within this window, a longer follow-up would better capture late recurrences and allow evaluation of anal fistula formation.

We did not perform extensive subgroup-specific performance analyses or apply explicit algorithmic fairness constraints. Inclusion criteria, data collection procedures, and modeling steps were applied uniformly across sociodemographic groups, and exploratory analyses suggested broadly similar performance by sex and age strata. Nevertheless, formal assessment of fairness and model performance across more diverse populations remains an important goal for future research.

Future work should proceed along several directions. The foremost priority is prospective and external validation of this nomogram across different healthcare settings and populations. Second, integrating molecular and microbiological data, for example through 16S rRNA sequencing of abscess microbiota, may help identify a “diabetic microbiome” signature predictive of recurrence and refine the model. Third, interventional studies are needed to determine whether implementing a risk-stratified management protocol guided by this nomogram can reduce recurrence rates and improve patient-reported outcomes. Such impact studies will be essential to establish the effectiveness and cost-effectiveness of the model in real-world clinical practice.

## 5 Conclusion

In conclusion, this study definitively identifies diabetes and suboptimal glycemic control as powerful, independent drivers of recurrence after surgery for perianal abscess. The developed and validated prediction model provides a clinically useful tool for risk stratification, facilitating the early identification of high-risk patients. Integrating routine HbA1c screening, aggressive perioperative glycemic management, and a personalized, risk-based follow-up strategy holds significant promise for reducing the clinical and economic burden of recurrent perianal disease. Our findings advocate for a paradigm shift toward a more metabolic-conscious and precision-based approach in the management of this common surgical condition.

## Protocol

No prospectively written or registered study protocol was prepared specifically for this retrospective observational analysis. The study procedures and statistical analysis plan followed institutional standards for clinical research and are described in detail in the Methods section. A full description of the protocol is available from the corresponding author on reasonable request.

## Registration

This prognostic modelling study was not registered in a public clinical-trial registry because it was a retrospective observational cohort based on routinely collected data and did not involve any experimental intervention.

## Patient and public involvement

Patients or members of the public were not involved in the design, conduct, reporting, or dissemination plans of this research.

## Data Availability

The raw data supporting the conclusions of this article will be made available by the authors, without undue reservation.
