# Correction: Impact of diabetes mellitus and glycemic control on postoperative recurrence of perianal abscess: a retrospective, single-center study

**DOI:** 10.3389/fmed.2026.1799950

**Published:** 2026-02-03

**Authors:** Frontiers Production Office

**Affiliations:** Frontiers Media SA, Lausanne, Switzerland

**Keywords:** diabetes mellitus, glycaemic control, glycated hemoglobin, HbA1c, nomogram, perianal abscess, prediction model, recurrence

Authors Tingting Li and Jianan Li were erroneously assigned as equal contributing authors. “^†^These authors have contributed equally to this work and share first authorship” has been removed.

The original version of this article has been updated.

